# Supplementary material for: Characteristics and outcomes of multisystem inflammatory syndrome in children: A multicenter, retrospective, observational cohort study in Mexico
Source: Front Pediatr. 2023 May 18;11:1167871. doi: 10.3389/fped.2023.1167871 (PMC10233130; doi:10.3389/fped.2023.1167871)
Supplement: Supplementary file 1 [file Table3.pdf]

Supplementary Chart 1. Variables included in Latent Class Analysis

. Any cardiac involvement: cardiac arrest OR any one or more of the following findings on Echo:
• Myocardial depression (left ventricular ejection fraction < 55%)
•Coronary aneurysms (coronary artery diameter z-score ≥ 2.5)
•Dilatation (z-score >2–<2.5)
• Mitral or aortic valve insufficiency
• Myocardial infarction
• Other abnormalities reported
2. Gastrointestinal involvement: Vomiting or diarrhea
3. Gastrointestinal involvement: Abdominal pain
4. Respiratory failure (any one of the following):
• CPAP
• Conventional ventilation
5. Kawasaki features: Conjunctivitis (bilateral, bulbar, non-suppurative)
6. Kawasaki features: Lymphadenopathy (cervical >1.5 cm diameter)
7. Kawasaki features: Rash (widespread, polymorphous, not vesicular)
8. Kawasaki features: Lips and mucosa (red cracked lips, strawberry tongue, erythematous oral cavity)
9. Kawasaki features: Changes of extremities (erythema, edema of palms and soles initially)
10. Any other rash
11. Hypotension
11. Albumin – low < 3g/dl
13. Raised ALT (>82 IU/L if <1yr, >56 IU/L if <3yrs, >58 IU/L if <7yrs, >72 IU/L if <13yrs, >74 IU/L if 13-15yrs)
14. Raised ferritin (>300 ug/L)
15. Raised D-dimers (>560 ng/ml)
16. Confusion
17. Creatinine (for <4yrs ≥78 umol/L, 5-11 years ≥106 umol/L, 12-15 years ≥180 umol/L)
18. Platelets (<150 x 10^3cells/mm^3)
19. Positive SARS-CoV-2 PCR test
20. Positive SARS-CoV-2 serology test
